# Gamma rays-assisted bacterial synthesis of bimetallic silver-selenium nanoparticles: powerful antimicrobial, antibiofilm, antioxidant, and photocatalytic activities

**DOI:** 10.1186/s12866-023-02971-1

**Published:** 2023-08-16

**Authors:** Reham R. El-Behery, El-Sayed R. El-Sayed, Gharieb S. El-Sayyad

**Affiliations:** 1 Drug Radiation Research Department, National Center for Radiation Research and Technology (NCRRT), Egyptian Atomic Energy Authority (EAEA), Cairo, Egypt; 2 Plant Research Department, Nuclear Research Center, Egyptian Atomic Energy Authority (EAEA), Cairo, Egypt

**Keywords:** Bimetallic Ag-Se NPs, Gamma-rays, Biological activity, Photo-catalysis, and *Candida albicans*

## Abstract

**Background:**

Bimetallic nanoparticles (BNPs) has drawn a lot of attention especially during the last couple of decades. A bimetallic nanoparticle stands for a combination of two different metals that exhibit several new and improved physicochemical properties. Therefore, the green synthesis and design of bimetallic nanoparticles is a field worth exploring.

**Methods:**

In this study, we present a green synthesis of silver nanoparticles (Ag NPs), selenium (Se) NPs, and bimetallic Ag-Se NPs using Gamma irradiation and utilizing a bacterial filtrate of *Bacillus paramycoides*. Different Techniques such as UV-Vis., XRD, DLS, SEM, EDX, and HR-TEM, were employed for identifying the synthesized NPs. The antimicrobial and antibiofilm activities of both the Ag/Se monometallic and bimetallic Ag-Se NPs were evaluated against some standard microbial strains including, *Aspergillus brasiliensis* ATCC16404, *Candida albicans* ATCC10231, *Alternaria alternate* EUM108, *Fusarium oxysporum* EUM37, *Escherichia coli* ATCC11229, *Bacillus cereus* ATCC15442, *Klebsiella pneumoniae* ATCC13883, *Bacillus subtilis* ATCC15442, and *Pseudomonas aeruginosa* ATCC6538 as a model tested pathogenic microbes. The individual free radical scavenging potentials of the synthesized Ag NPs, Se NPs, and bimetallic Ag-Se NPs were determined using the DPPH radical scavenging assay. The degradation of methylene blue (MB) dye in the presence of the synthesized Ag NPs, Se NPs, and bimetallic Ag-Se NPs was used to assess their photocatalytic behavior.

**Results:**

According to the UV-Vis. spectrophotometer, the dose of 20.0 kGy that results in Ag NPs with the highest O.D. = 3.19 at 390 nm is the most effective dose. In a similar vein, the optimal dose for the synthesis of Se NPs was 15.0 kGy dose with O.D. = 1.74 at 460 nm. With a high O.D. of 2.79 at 395 nm, the most potent dose for the formation of bimetallic Ag-Se NPs is 15.0 kGy. The recorded MIC-values for Ag-Se NPs were 62.5 µg mL^− 1^, and the data clearly demonstrated that *C. albicans* was the organism that was most susceptible to the three types of NPs. The MIC value was 125 µg mL^− 1^ for both Ag NPs and Se NPs. In antibiofilm assay, 5 µg mL^− 1^ Ag-Se NPs inhibited *C. albicans* with a percentage of 90.88%, *E. coli* with a percentage of 90.70%, and *S. aureus* with a percentage of 90.62%. The synthesized NPs can be arranged as follows in decreasing order of antioxidant capacity as an antioxidant result: Ag-Se NPs > Se NPs > Ag NPs. The MB dye degradation in the presence of the synthesized Ag NPs, Se NPs, and bimetallic Ag-Se NPs was confirmed by the decrease in the measured absorbance (at 664 nm) after 20 min of exposure to sunlight.

**Conclusion:**

Our study provides insight towards the synthesis of bimetallic NPs through green methodologies, to develop synergistic combinatorial antimicrobials with possible applications in the treatment of infectious diseases caused by clinically and industrial relevant drug-resistant strains.

## Background

Different metallic nanoparticles have been created and identified during the past several decades as a result of their unique features and the many applications they have in optoelectronics, biological sensors, biological imaging, catalytic processes, antibacterial, and different biological fields [1–5].

Due to the unique chemical, physical, and physiological characteristics of bimetallic nanomaterials (BMNMs), which are the consequence of the synergistic effects of combining diverse metallic components, they have attracted a lot of attention [6, 7]. Gold-silver nanoparticles (Au-Ag NPs) composites have received the most attention among the various BMNMs because of their adaptable plasmonic properties that rely on their structural makeup and their potential for use in sensor and catalytic activities [8]. The combined reduction of sodium selenite and silver nitrate precursors in the incidence of gamma rays is one of several known preparation techniques for the creation of bimetallic Ag-Se NPs, as is the use of some biological filtrates as an especially powerful stabilizing agent.

During the manufacture of inorganic-polymer nanoparticles, natural polymeric protective coatings including chitosan, Gum Arabic, and Polyvinylpyrrolidone (PVP), as well as certain bacterial filtrate, are utilized to boost the resilience of the generated NPs and to manage their particle size [9, 10].

The interactions between living cells and some particles are now included in biological techniques to nanoparticle and nanocrystal production [11]. Due to its safety and environmental friendliness, biological production of metal nanoparticles utilizing plants, bacteria, and fungus has recently attracted a lot of attention [12]. According to earlier research, bacterial extracts include macromolecules such phenolics, flavonoids, alkaloids, polysaccharides, proteins, enzymes, and tannins that work as safe reducing and stabilizing agents during the production of metal nanoparticles [13].

Many commercial uses, including surface sprays, healthcare equipment, refrigerators, antibacterial substances, and several more, may be made with silver and selenium nanoparticles (NPs) [14, 15]. The capacity of Ag NPs to inhibit microbial development is well established. But since the methods effectiveness and their damaging impact on the target cells remain not completely understood, more study is necessary [15, 16].

The microbial activity of multi-drug resistant bacteria and other infectious microorganisms linked to diabetic foot are endangering the health of the general people. One of these microorganisms’ most prevalent characteristics is microbial biofilm, which is a collection of multicellular organisms that form protective layers surrounding the diseased diabetic foot [17, 18]. It kept the microbial population alive in almost all damp settings with limited nutrient flow [19]. Most pathogenic bacteria develop an exopolysaccharide biofilm around their whole population as a protective barrier against a variety of bacterio-phages, biocides, and immune cells from the host in order to survive harsh environmental and other circumstances [17, 18].

Due to either the limited quantity of drinkable water that is now available on Earth (approximately 0.9%), or the enormous amount of wasted or contaminated water, the world is currently experiencing a worldwide water shortage crisis [20, 21]. Furthermore, many harmful bacteria that cause severe illnesses including hepatitis A, diarrhoea, and typhoid fever thrive in dirty water [22, 23]. Therefore, it is important now to create innovative technology for wastewater treatment [24, 25]. There are now several water treatment techniques, which may be divided into three categories: processes that are physical, chemical, and biological [26, 27]. One of these, heterogeneous photocatalysis using nanoscale semiconductor photocatalysts, is a quick, effective, and affordable way to purify water [26, 28–30]. This method depends on redox processes that happen on the semiconductor photocatalyst surfaces when they absorb light with a greater energy than their bandgap [31–33].

Bacterial filtrate was used as a promising eco-friendly and economically advantageous material for the controlled synthesis of Ag NPs, Se NPs, and bimetallic Ag-Se NPs with the influence of gamma radiation. Optimization was applied to get precise and superior results in terms of the purity, shape, and size of the synthesized NPs. A variety of pathogenic bacteria and yeasts were tested for the generated NPs’ antimicrobial and antibiofilm activities. In addition, the antioxidant potential and photocatalytic behavior of the synthesized NPs had been investigated to direct the synthesized NPs to be utilized in different biomedical, and industrial applications due to their promising activities.

## Materials and methods

### Chemicals and reagents

For the fabrication of NPs, analytical-grade chemicals including sodium selenite (Sigma Aldrich, UK) and silver nitrate (Sigma Aldrich, UK) were used. However, media for microbiological testing was acquired from Oxoid in the UK.

### Source of bacteria

*Bacillus paramycoides* strain (registered in Gene bank with accession no MT102429 [34]), supplied from Drug Microbiology Lab., culture collection of the Drug Radiation Research Department at the National Center for Radiation Research and Technology (NCRRT), EAEA, Cairo, Egypt, was employed for the Ag NPs, Se NPs, and bimetallic Ag-Se NPs production.

### Bacterial filtrate preparation

Every two weeks, seed media was used to continuously sub-cultivate the culture. *Bacillus paramycoides* was grown in a 250 mL Erlenmeyer flask at 30^o^C with 200 rpm shaking for 24 hours using (LAB-Line Orbit Environ) in 50 mL medium (fermentation media for nitrate reductase production) containing the following concentrations of yeast extract (0.3%), peptone (0.5%), and K NO_3_ (0.2%). Using a Hettich Universal 16R cooling centrifuge set at 6000 rpm for 10 minutes (6^o^C), the cell-free supernatant was separated [35].

### Gamma radiation

The NCRRT in Cairo, Egypt, performed gamma irradiation procedures. The collected samples were gamma-irradiated in the form of solutions employing the ^60^Co-Gamma chamber 4000-A-India after the first precursors were dissolved at a radiation time that was predicted to be 1.014 kGy per hour (dose rate).

### Synthesis of Ag NPs, Se NPs and bimetallic Ag-Se NPs

With bacterial filtrate (as a reducing and capping agent) and gamma rays (as a direct and indirect reducing processing), Ag NPs, Se NPs, and bimetallic Ag-Se NPs were created. Because powerful reducing electrons, known as e^-^_aq_, were unleashed by gamma rays and were present in aqueous solutions, this process of direct reduction of metal ions was triggered [36]. While indirect reduction came about as a result of the radiolysis byproducts H^•^ and OH^•^ interacting with the bacterial filtrate to produce active free radical, which then reduced metal ions [37].

Aqueous bacterial filtrate solution was combined with mixed solution samples of (1.0 mM) Ag NO_3_ for the production of Ag NPs. Similar to this, numerous solution samples of sodium selenite (1.0 mM) were combined with bacterial filtrate to create Se NPs. While multiple solution samples of (1.0 mM) Ag NO_3_ and (1.0 mM) sodium selenite were combined with bacterial filtrate to create bimetallic Ag-Se NPs. Prior to gamma radiation, the pH of each sample was determined and adjusted to neutral (pH = 7). Then, various dosages of gamma radiation (0, 5.0, 10.0, 15.0, 20.0, and 25.0 kGy) were applied to the prepared solutions. In order to determine the most efficient dose, the optical density (O.D.) of the produced NPs at a specific and defined wavelength was determined using UV-Vis. spectra. In the case of bimetallic Ag-Se NPs, in addition to dose, the ratio of Ag/Se concentration must be explored as a critical influence [38, 39].

### Characterization of the synthesized NPs

The absorbance and optical characteristics of the synthesized Ag NPs, Se NPs, and bimetallic Ag-Se NPs were investigated using a UV-Vis. spectrophotometer (JASCO V-560). A sample without any metallic ions was added for auto-zero purposes. To determine the fixed wavelengths needed to compute absorbance, all samples were first tested for optical properties [40].

A high-resolution transmission electron microscope (HR-TEM, JEM2100, Jeol, Japan) was also used to examine the shape, appearance, and average particle size of the generated NPs. After dried in an incubator at 37.0 ± 2 °C, NPs samples utilized for TEM research were drop-coated into carbon-coated TEM grids. The XRD-6000 (Shimadzu Scientific Instruments, Japan) uses XRD analysis to confirm the accurate development of the crystalline materials. XRD analysis was used to estimate the crystal size in the resulting bimetallic NPs. The last stage comprised analyzing the deposited bimetallic elements composition using an EDX detector (JEOL JSM-5600 LV, Japan) and analyzing the surface quality and exact surface form of the synthesised bimetallic NPs using a SEM, ZEISS, EVO-MA10, Germany [41].

### Separation and purification of the synthesized NPs

The produced Ag NPs, Se NPs, and bimetallic Ag-Se NPs were separated from the reaction mixture and purified using Whatman No. 1 filter paper. Each kind of NPs was obtained by ultracentrifugation at 20,000 rpm for 20 min, followed by ethanol and deionized water washings and drying at 50 °C. Each NP type’s resulting fine powders were then individually dissolved in HPLC-grade ethanol, given an ultrasonic treatment to disperse them, and then utilized for the biological assessment [42].

### In vitro antimicrobial sensitivity tests

To acquire a concentration range of 62.5–1000 µg mL^− 1^, Ag NPs, Se NPs, and bimetallic Ag-Se NPs were individually dissolved in various quantities of HPLC-grade methanol. After that, they were subjected to an ultrasonic treatment. The agar well diffusion assay approach was used to assess antimicrobial activity [43]. *Aspergillus brasiliensis* ATCC16404 and *Candida albicans* ATCC10231, two human pathogenic fungi, and *Alternaria alternate* EUM108 and *Fusarium oxysporum* EUM37, two plant pathogenic fungi, were tested for antifungal potential by Cairo MIRCEN (Faculty of Agriculture, Ain Shams University, Cairo, Egypt). The agar wells were also treated simultaneously with Nystatin (positive control) and only methanol (negative control).

The antibacterial susceptibility experiment was conducted in the meanwhile using some harmful bacterial strains, including *Escherichia coli* ATCC11229, *Bacillus cereus* ATCC15442, *Klebsiella pneumoniae* ATCC13883, *Bacillus subtilis* ATCC15442, and *Pseudomonas aeruginosa* ATCC6538. Amoxicillin/Clavulanic acid (positive control) and methanol alone (negative control) were applied to the agar wells to create control Petri plates. The agar wells’ inhibition zones were meticulously measured. The lowest concentration of the synthesized NPs with the greatest level of inhibition, known as the MIC (minimum inhibitory concentration), was found in agar wells.

### Anti-biofilm potential of the prepared NPs

By using the technique described by Christensen et al., a semi-qualitative assessment of biofilm development was made [44]. It was easy to see the biofilm wrapping on the inner surfaces of test tubes absent of Ag NPs, Se NPs, and bimetallic Ag-Se NPs. Pathogenic microbes that demonstrated responsiveness in the antimicrobial experiment were used to determine the antibiofilm properties of synthesized Ag NPs, Se NPs, and bimetallic Ag-Se NPs (at 5.0 µg/mL) and evaluate it to the control tube. The examined bacteria and *Candida* sp. broth were given treatment (after adjustment at 0.5 McFarland) and added to each tube’s 5.0 mL of nutrition broth before being incubated at 37 °C for an overnight period [45].

The adhering bacteria and yeast that had covered the inner walls of test tubes for 15 min were rinsed with roughly 5.0 mL of sodium acetate (3.5%). Then, they were washed using de-ionized water (D.I.W). About 5.0 mL of crystal violet (CV; 0.15%) was employed for staining microbial biofilms for 15.0 min before the remaining dye was removed. After that, the color was dissolved using 5.0 mL of 100% ethanol [45]. If a noticeable discolored film covering the tube’s inside surfaces was discovered, the biofilm that had been generated was identified [46]. The microbial biofilms were investigated using a UV-Vis. spectrophotometer at a fixed wavelength (570.0 nm). The bacterial and yeast biofilm inhibition (%) was used for calculating the inhibition % as the following: [44].

Inhibition % = 100× (O.D. of control sample – O.D. of treated sample) / (O.D. of control sample).

### In vitro antioxidant activity

The 2, 2-diphenyl picrylhydrazyl (DPPH, Sigma-Aldrich, St. Louis, MO, USA) radical scavenging experiment was used to determine the individual free radical scavenging potentials of the synthesized Ag NPs, Se NPs, and bimetallic Ag-Se NPs [43]. Each NPs were independently diluted in HPLC-grade methanol and subjected to an ultrasonic treatment to obtain concentrations between 25 and 1000 µg mL^− 1^. The antioxidant standard ascorbic acid from Sigma-Aldrich, was used as a control substance at the same dose range [47]. The observed absorbance difference between the combination (DPPH + NPs) and the control (DPPH alone) was used to calculate the percentage of scavenging activity. GraphPad Prism software, San Diego, CA, USA, was used to determine IC50 values from graphic plots for each NPs concentration.

### Photocatalytic potential

According to the procedure outlined by Abdelhakim et al. [48], the breakdown of methylene blue (MB) dye (Sigma-Aldrich, USA) was used to test the photocatalytic efficiency of the synthesized Ag NPs, Se NPs, and bimetallic Ag-Se NPs. Different quantities of both Ag NPs, Se NPs, or bimetallic Ag-Se NPs powder (25, 50, 100, 200, and 400 mg) were separately added to a 100 mL aqueous solution of MB (10 mg L^− 1^) while stirring constantly for an hour in full darkness in order to attain the adsorption equilibrium. The combination was then exposed to sunlight for 20 min at the ambient temperature. Following that, 10 mL of the aliquot solution from the NPs-dye combination was removed, centrifuged, and the absorbance was measured at 664 nm. Under the same circumstances, a control experiment was conducted without the inclusion of nanoparticles. The following equation was used to determine the percentage of dye degradation:

Degradation (%) = 100 × (Mo - M) / Mo.

*Where Mo is the original concentration of MB and M is the MB concentration after catalytic degradation.*

### Statistics

The computed mean and standard deviation were used to express the experimental results. The estimated mean is based on three separate experiments’ worth of triplicate readings. The least significant difference (LSD) test (0.05 level) and one-way analysis of variance (ANOVA) were used to examine statistical significance using IBM Corp.‘s SPSS software, version 22.

## Results and Discussion

### Synthesis of Ag NPs, Se NPs and bimetallic Ag-Se NPs by bacterial filtrate and gamma rays

#### The impact of gamma radiation and the ideal dosage

**Fig. 1:**
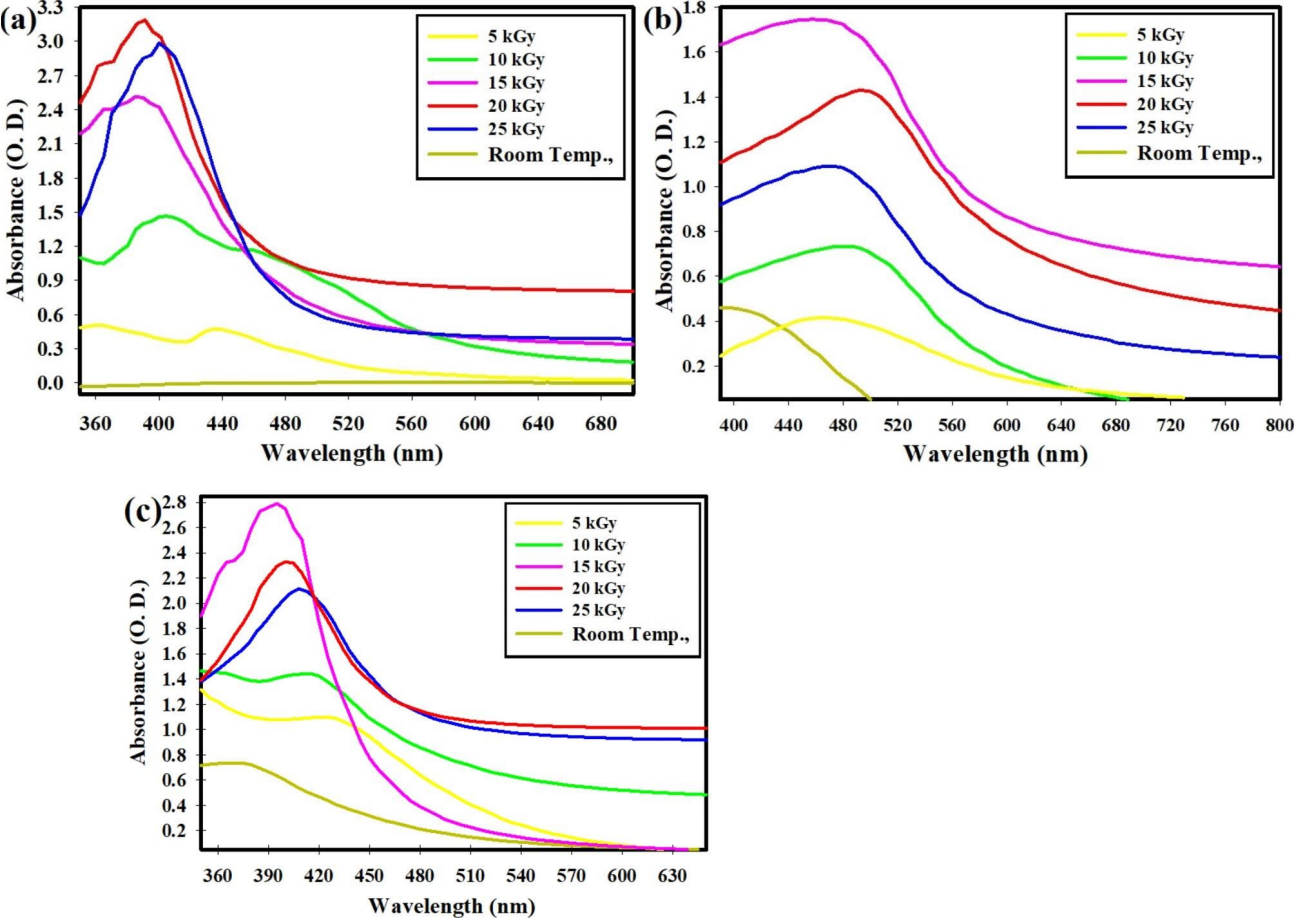
Gamma-rays screening by UV-Vis. spectrophotometry for the synthesis of Ag NPs **(a)**, Se NPs **(b)**, and bimetallic Ag-Se NPs **(c)**

Figure 1 displays gamma-rays screening for the created NPs applying a UV-Vis. spectrophotometer. The most effective dosage is shown (Fig. 1a) to be 20.0 kGy, which results in Ag NPs production with the highest O.D. = 3.19 at 390 nm. Similar to this, Fig. 1b shows that the optimal dose for producing Se NPs is 15.0 kGy with O.D. = 1.74 at 460 nm. While Fig. 1c demonstrates that 15.0 kGy, with a high O.D. of 2.79 at 395 nm, is the most potent dose for the formation of bimetallic Ag-Se NPs.

In this case, extreme temperatures or additional chemical reducing agents were not required to make homogeneous NPs with a substantial comparative yield thanks to the potential of gamma rays [49, 50].

Figure 1 shows that the maximum Gamma-ray dose used to produce Ag NPs, Se NPs, and bimetallic Ag-Se NPs, respectively, was 20 kGy, 15 kGy, and 15 kGy. After raising the dose by more than 15 kGy (in the case of Se NPs and Ag-Se NPs) and more than 20 kGy (in the case of Ag NPs), the relative yield of the synthesized NPs had decreased. Excessive radiation is not helpful for the creation of new nanoparticles due to extra reactive oxygen species and solvated electrons (produced by water radiolysis) alter the pH of the solutions, attack freshly established NPs (which have contrary charges), attach to them, and ultimately generate combined NPs that lower the intensity in the UV-Vis. spectrum.

### Proposed synthetic reaction mechanism

Investigations into kinetics show that the onset of gamma irradiation always precedes the start of metal ions’ conversion to NPs in the prepared solutions. According to Table 1, the reduction in the present investigation was greatest at 20.0 and 15 kGy, indicating that gamma radiation is required for the synthesis of Ag NPs, Se NPs, and bimetallic Ag-Se NPs [51].

**Table 1:** Proposed reaction mechanism regarding Ag NPs, Se NPs, and bimetallic Ag-Se NPs synthesis

Reaction inputs	Condition	Products	Eq.
H_2_O	Radiolysis (γ-ray)	e^−^ _aq_, OH^•^, H^•^, H_2_,and H_2_O_2_	(1)
Ag NO_3_ + H_2_O	Hydrolysis	Ag^+^ + NO_3_^−^	(2)
Na_2_SeO_3_ + H_2_O		SeO_3_ ^− 2^ + 2Na^+^	(3)
Ag^+^ + e^−^ _aq_	Reduction	Ag NPs	(4)
SeO_3_ ^− 2^ + e^−^ _aq_		Se NPs + 3O_2_	(5)
SeO_3_ ^− 2^ +3Ag^+^	Complexation	Ag-Se NPs + 3O_2_	(6)

After being exposed to gamma radiation, water created a variety of species, including e^−^ _aq_, OH^•^, H^•^, H_2_O_2_, and H_2_, according to Eq. (1). The creation of highly reducing electrons, or e^−^ _aq_, which carry out their task without generating any unnecessary byproducts was a benefit of gamma irradiation for the manufacture of metallic nanoparticles [14]. Different NPs were created starting with the breakdown of Ag NO_3_ and Na_2_SeO_3_, which resulted in the hydrate cations Na^+^ and Ag^+^ and the anions SeO_3_^2−^ and NO_3_^−^ (Eqs. (2) and (3) in Table 1) [52].

After then, according to Eqs. (4) and (5), there is a chance that both Ag^+^ and SeO_3_^2−^ will be obviously decreased by e^−^ _aq_, creating non-capped Ag NPs and Se NPs that are likely to disintegrate [53]. In simultaneously as shown in Eq. (6) (Table 1), Ag^+^ and SeO_3_^2−^ likely reacted to create the synthesized bimetallic Ag-Se NPs combination [54].

A phenomenon known as Surface Plasmon Resonance (SPR) is brought about by the stimulation of electrons in the conductive zone around bimetallic Ag-Se NPs [55]. The unique oscillation qualities depend on the particle’s size and form. It is significant to note that when inorganic NPs are activated by a light source, light electromagnetism bridges the free electrons, namely those that conduct line-located electrons of Ag ^+^ and/or Se ^+^ ions to create fused mixed flow [56].

The whole process demonstrated how Ag^+^ and SeO_3_^2−^ ions were reduced by electrons to allow for the formation of bimetallic Ag-Se NPs. It should be observed that the mean size of the particles and particle size distribution of the generated Ag NPs, Se NPs, and bimetallic Ag-Se NPs increased when the gamma radiation dosage was increased up to 20.0 kGy [57], result was attributed to the agglomeration and deposition of the synthesized Ag NPs, Se NPs, and bimetallic Ag-Se NPs via the mechanism shown in Eq. (1), which was influenced by additional electrons and free radicals produced during water radiolysis [58].

### Characterization of Ag NPs, Se NPs and bimetallic Ag-Se NPs

#### HR-TEM imaging, and DLS analysis

**Fig. 2:**
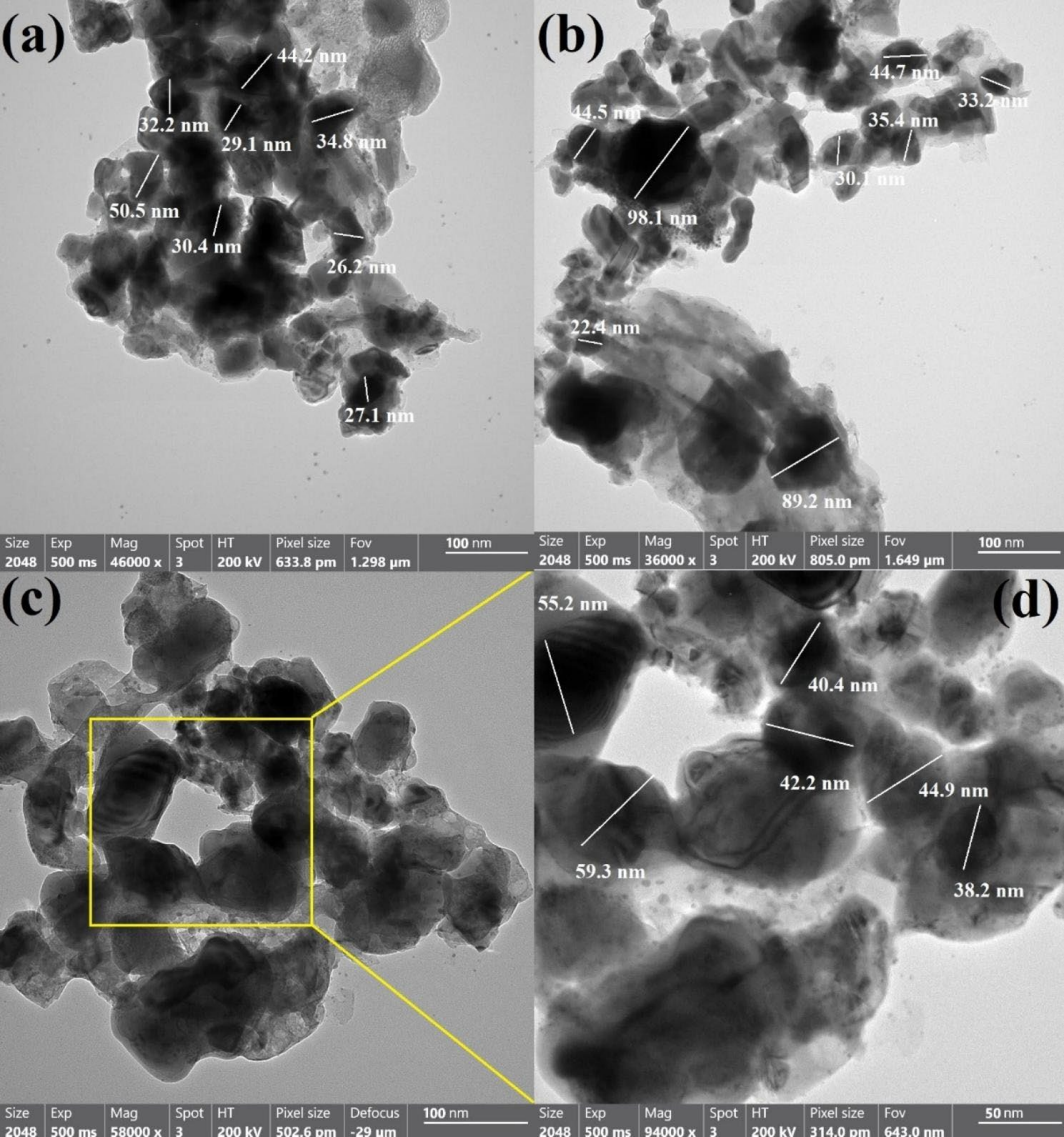
Average particle size, and shape for the synthesized NPs where, **(a)** HRTEM for Ag NPs, **(b)** HRTEM for Se NPs, **(c)** HR-TEM for bimetallic Ag-Se NPs, and **(d)** magnified HR-TEM for bimetallic Ag-Se NPs

The average particle size and the appearance of the synthesized Ag NPs, Se NPs, and bimetallic Ag-Se NPs were examined by HR-TEM (Fig. 2). Results from HR-TEM and DLS measures were also compared. Various spherical, sometimes irregular, and oval shapes of the synthesized Ag NPs, Se NPs, and bimetallic Ag-Se NPs could be seen in HR-TEM images. According to Fig. 2a, the diameter of Ag NPs ranged from 26.2 nm to 50.5 nm with an average of 34.3 nm. As shown in Fig. 2b, Se NPs ranged in size from 22.4 to 98.1 nm, with a median diameter of 40.7 nm. Finally for the synthesized bimetallic Ag-Se NPs, the particle sizes ranged from 38.2 nm to 59.3 nm with an average size as 46.7 nm (Fig. 2c, and d).

According to the HRTEM image result (Fig. 2d), there was only one grade system since the line spacing was precisely the same throughout. It shown that the selenium matrix’s homogeneous distribution of silver led to the creation of a special alloy. Similar to this, the bacterial filtrate’s generated radical-multi-position may cause concurrent decreases in Ag and Se [57].

The anisotropic form had been identified, although different morphologies may have been seen as a result of the process of creating it from extract in that present work [59]. This is due to the collected NPs were almost always round or oval in form. Due to the use of a single reducing and capping agent, a constant form is seen in our investigation. Last but not least, our findings were connected to the newly published studies [60–63].

**Fig. 3:**
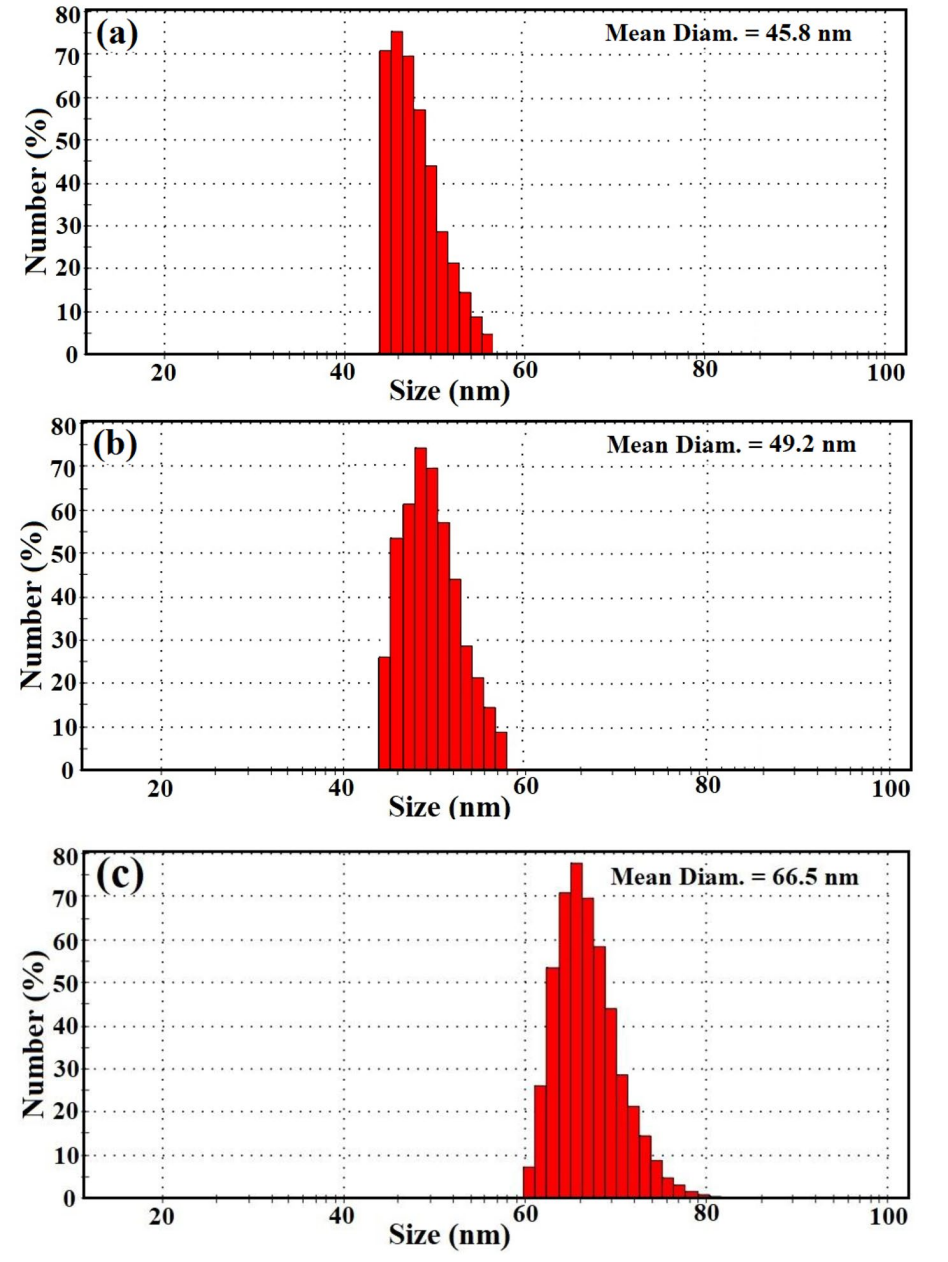
Particle size distribution of **(a)** Ag NPs, **(b)** Se NPs, and **(c)** bimetallic Ag-Se NPs by DLS analysis

The usual particle size spreading for Ag NPs, Se NPs, and bimetallic Ag-Se NPs, which were produced using gamma rays and bacterial filtrate, was strongminded by the DLS technique to be 45.8 nm, 49.2 nm, and 66.5 nm, respectively (Fig. 3a, b, and 3c).

When the polydispersity index (PDI) results are less than 0.05, samples are said to be monodisperse by international standards organizations (ISOs). The goal of PDI results larger than 0.7, however, is to create particles with a polydispersity distribution [64].

According to our research, the bimetallic Ag-Se NPs had PDI values of 0.86. The synthesized bimetallic nanoparticles were a reasonable range of polymers based on the existing values. The results demonstrated that the estimated particle sizes detected by HR-TEM imaging were lower than the median and common sizes suggested by DLS analysis. The large diameters of the synthesized NPs are caused by the hydrodynamic radius within the Ag NPs, Se NPs, and bimetallic Ag-Se NPs as well as the water layers encircling them [65].

#### SEM and EDX analysis

**Fig. 4:**
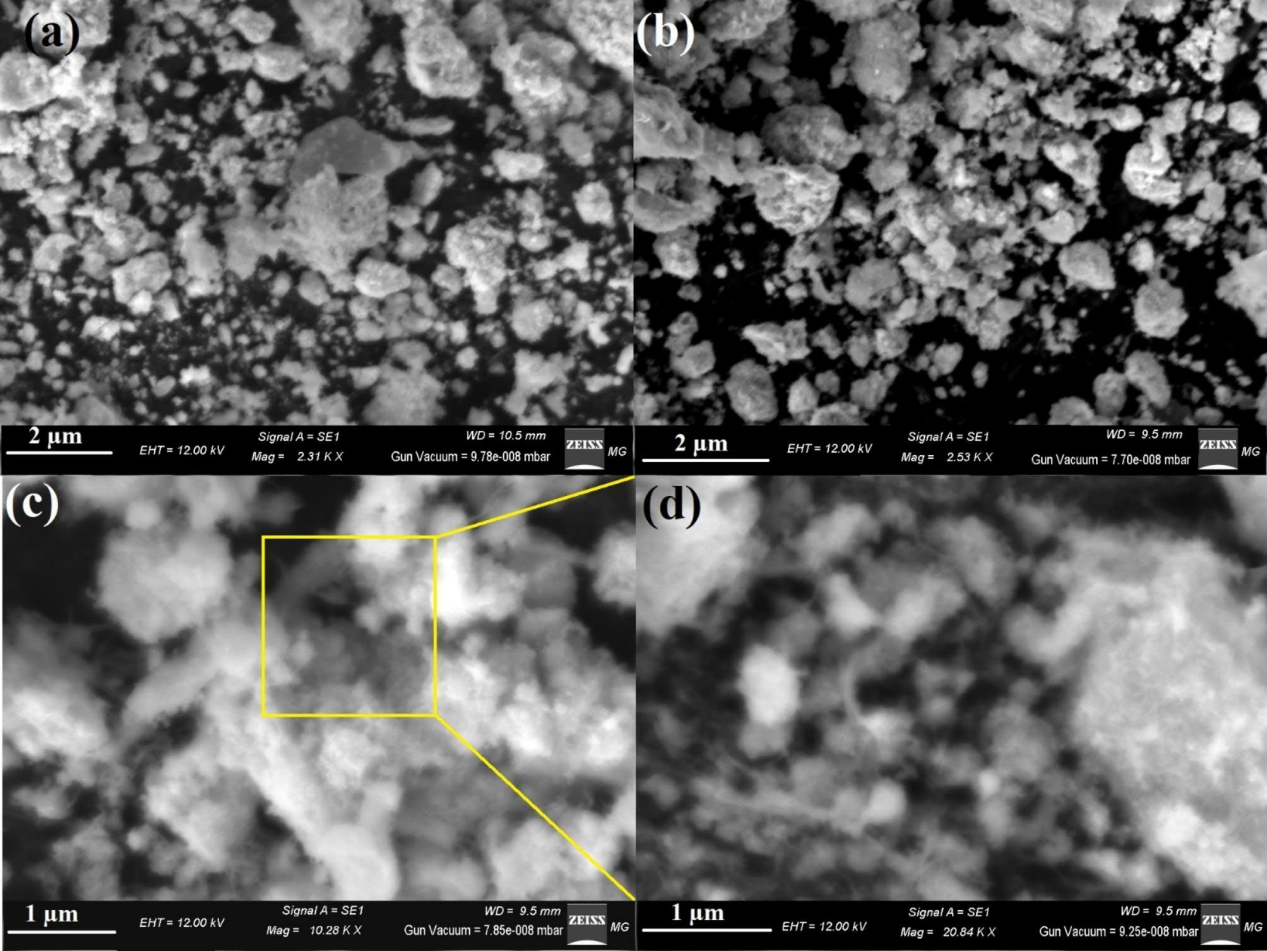
Morphological characters and surface shape for the synthesized NPs where, **(a)** SEM for Ag NPs, **(b)** SEM for Se NPs, **(c)** SEM for bimetallic Ag-Se NPs, and **(d)** magnified SEM for bimetallic Ag-Se NPs

Figure 4 depicts the surface morphology of the synthesized Ag NPs, Se NPs, and bimetallic Ag-Se NPs. The image in Fig. (4a) shows that Ag NPs, which are visible as a brilliant particle, were frequently in their pure state. Similar to how Se NPs appeared as brilliant particles all over the carbon imaging holder in Fig. (4b).

Figure (4c) displays the SEM verification of the synthesized bimetallic Ag-Se NPs, which were dispersed equally across the carbon visualization frame. The synthesized bimetallic Ag-Se NPs, however, appeared to be just one particle with an irregular surface shape when seen in high resolution (Fig. 4d).

By comparing the synthesized Ag NPs, Se NPs, and bimetallic Ag-Se NPs (in the present research) to the literature’s morphology form and elemental analysis, it was found that they were equally distributed with small size and a comparable spherical shape.

The synthesized bimetallic Au-Ag NPs were created by Muhammad et al. [66] using the citrate reduction technique at various pH levels and temperatures. Since their apparent morphology form and border size showed that they fall within a size range of 50 nm to 65 nm and appeared as spheres, temperature and pH are significant elements in the production process.

**Fig. 5:**
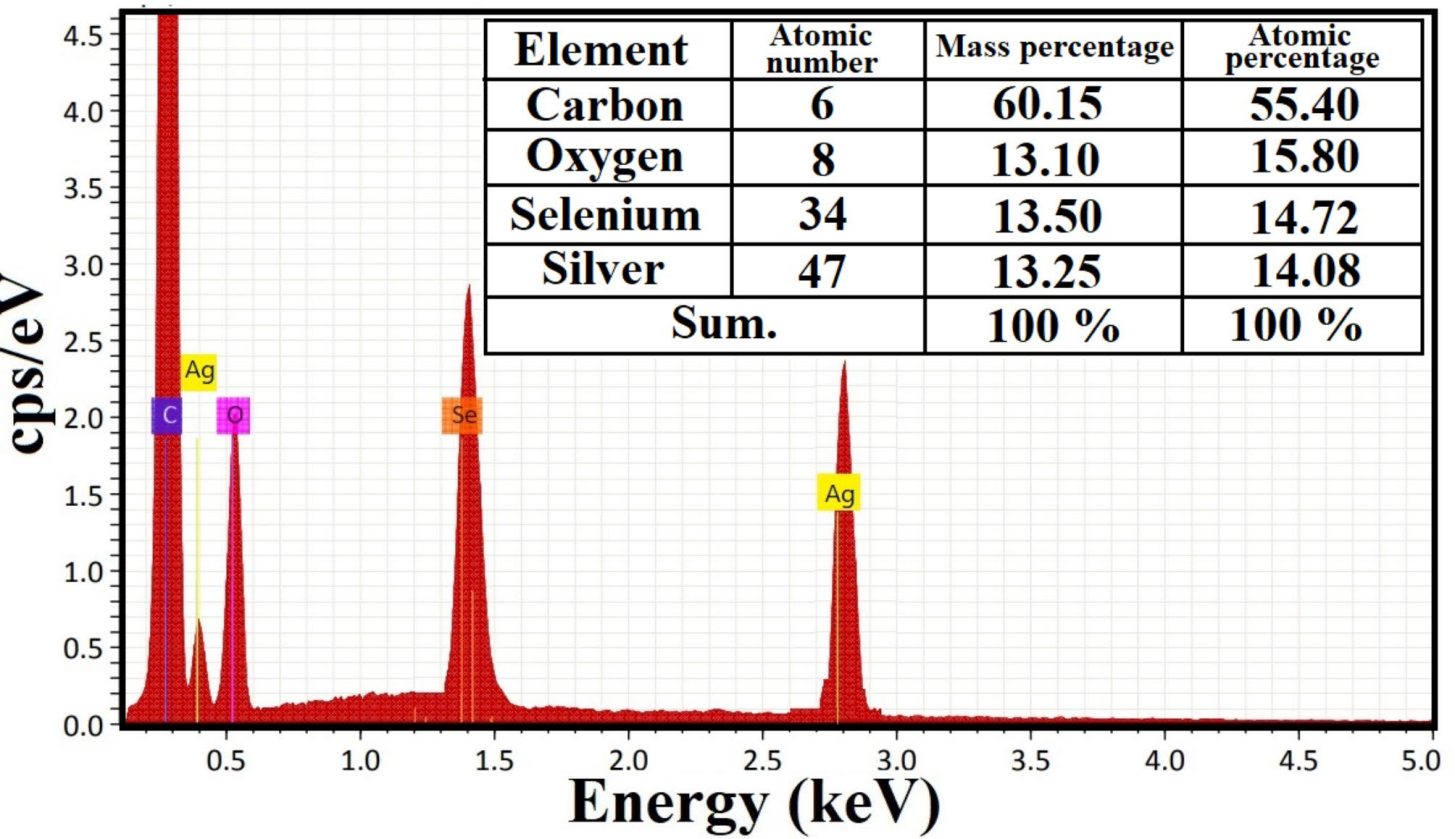
Elemental investigation for the synthesized bimetallic Ag-Se NPs using EDX analysis

To examine the basic composition and confirm the percentage variations of the generated samples, EDX spectroscopy was used [67]. As shown in Fig. 5, the purity and fundamental structure of the synthesized bimetallic Ag-Se NPs were determined using an EDX examination. Bimetallic Ag-Se NPs showed distinct silver and selenium absorption maxima at 1.41 and 2.85 keV, respectively. The Ag-Se NPs’ element purity is supported by the absence of other elements peaks and the abundance of Ag and Se in the spectrum, while O and C signals were for the image holder, as shown in Fig. 5.

The mass percentage was found to be particularly equal in case of Ag (13.25%), and Se (13.50%) and confirm the equal distribution of Ag, and Se elements in the synthesized bimetallic Ag-Se NPs (Fig. 5).

Ag-Au bimetallic NPs’ elemental structure was analyzed by EDX and contrasted with that of Muhammad et al. [66], it demonstrates that Ag and Au are used to create bimetallic NPs. At 25 °C, Au’s relative elemental percentage was 55.98% and Ag’s was 44.02%; at 100 °C, these percentages shifted to Au (69.51%) and Ag (30.49%), demonstrating the effectiveness of the reduction process.

#### XRD investigation

**Fig. 6:**
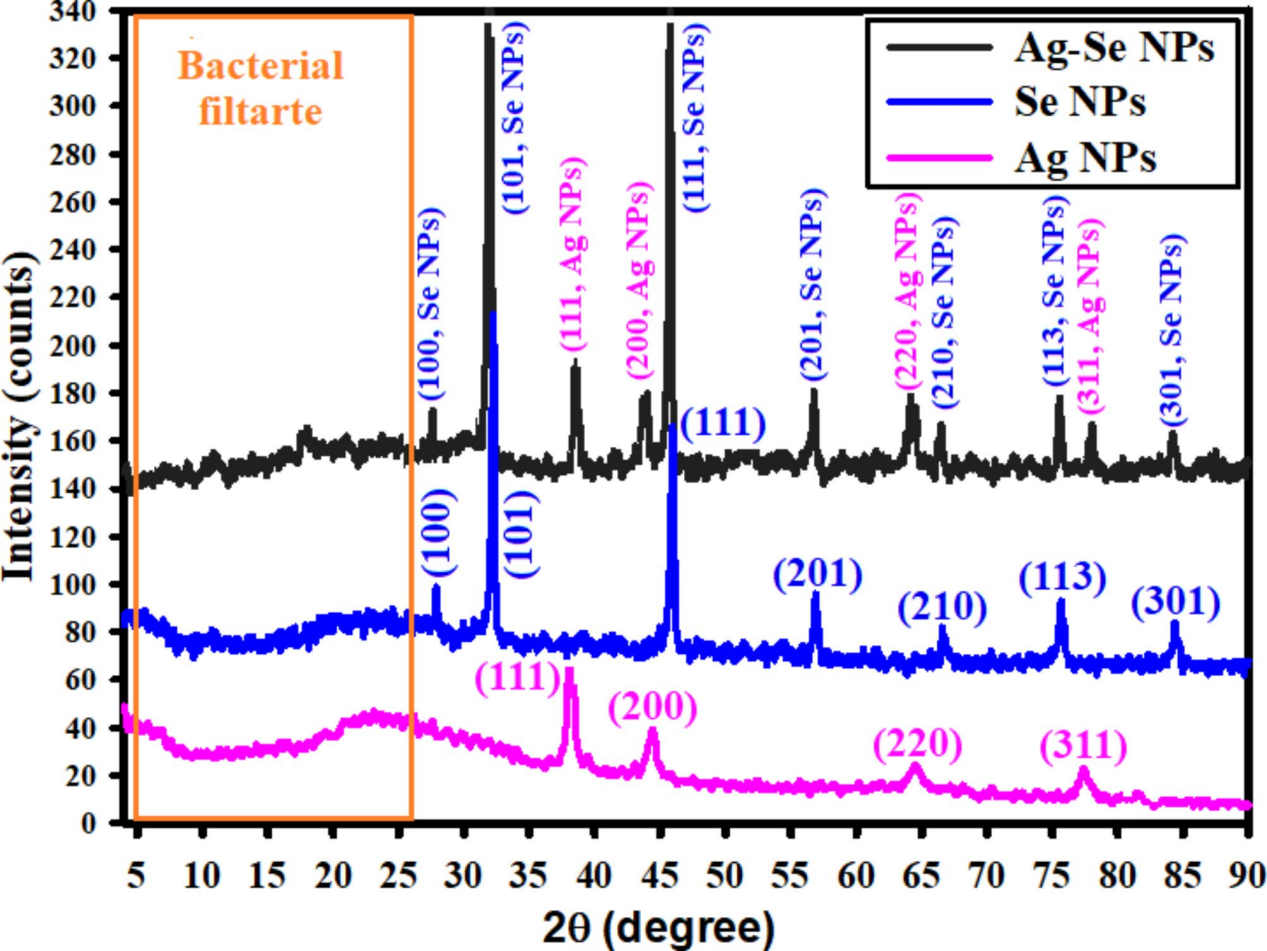
XRD spectra for the synthesized Ag NPs, Se NPs, and bimetallic Ag-Se NPs

The structure of the crystals and phase of the generated NPs were investigated using a technique called XRD [68]. Figure 6 displays the synthesized NPs’ XRD pattern. The pattern makes it very evident that neither sodium selenite nor silver nitrate exhibit any distinctive peaks. The production of the nano-complex (bimetallic Ag-Se NPs) was verified by XRD measurements. Figure 6 shows the XRD diffraction peaks of Ag NPs, including peaks at 2 ɵ = 38.46^o^, 44.14^o^, 64.88^o^, and 78.61^o^ that correspond to (111), (200), (220), and (311), respectively for Bragg’s reflections and matched with a reference card JCPDS-ICDD card 04-0783 [69]. Additionally, Fig. 6 displays the XRD diffraction peaks of Se NPs and displays the diffraction characteristics for 2 ɵ at 27.24^o^, 33.19^o^, 46.88^o^, 57.29^o^, 67.19^o^, 75.07^o^, and 84.68^o^, which correspond to the Bragg’s reflections at (100), (101), (111), (201), (210), (113), and (301), respectively. Using a standard card JCPDS File No. 06-0362, the Joint Committee on Powder Diffraction Standards (JCPDS) of Se NPs demonstrated that all of the peaks were equivalent [70].

Bimetallic Ag-Se NPs that have been synthesized have diffraction properties at 2 ɵ that are similar to those seen for both Se NPs and Ag NPs, according to XRD data. This suggests that the synthesized bimetallic Ag-Se NPs were crystallized. It is important to note that a minor in 2 ɵ shifting was found, which may be related to the emergence of bimetallic Ag-Se NPs [68]. According to the published experiment [71], the 2 ɵ range from 5^o^ to 25^o^ corresponded to the amorphous form of organic molecules found in the bacterial filtrate.

The lack of peaks at 2 ɵ = 31.30^o^, 32.62^o^, and 33.68^o^ demonstrated the purity and absence of AgO NPs and SeO NPs in the synthesized Ag NPs, Se NPs, and bimetallic Ag-Se NPs [72]. According to the XRD data, the generated nanoparticles were extremely crystalline for improved applicability [73]. On the other hand, the average crystallite size of the synthesized Ag NPs, Se NPs, and bimetallic Ag-Se NPs was determined by using the Scherrer equation [74], and was found to be 30.25, 38.54, and 42.40 nm for the synthesized Ag NPs, Se NPs, and bimetallic Ag-Se NPs, respectively. Similarly, the assessed crystal sizes detected by XRD data and after applying the Scherrer equation were lower than sizes investigated by DLS analysis. The large diameters of the synthesized NPs are caused by the hydrodynamic radius within the Ag NPs, Se NPs, and bimetallic Ag-Se NPs as well as the water layers surrounding them [65, 75].

### In vitro antimicrobial activity of the synthesized Ag NPs, Se NPs and bimetallic Ag-Se NPs

The antifungal activity of the synthesized Ag NPs, Se NPs, and bimetallic Ag-Se NPs against two fungi that are harmful to humans and two fungi that are harmful to plants were shown in Table 2. When compared to the standard antifungal (Nystatin), all the synthesized NPs demonstrated promising antifungal potential. The data collected (Table 2) further showed that the MIC-values of the three types of NPs vary depending on the fungal pathogen examined as well as depending on the synthesized NPs. Data clearly demonstrated that *C. albicans* was the most susceptible organism to all three kinds of NPs, with documented MIC values for bimetallic Ag-Se NPs of 62.5 µg mL^− 1^. Ag NPs and Se NPs both have MIC values of 125 µg mL^− 1^, however shown in Table 2.

**Table 2:** Antifungal activity of Ag NPs, Se NPs, and bimetallic Ag-Se NPs synthesized by bacterial filtrate and gamma-rays against different human and plant pathogenic fungi

NPs concentration (µg mL^− 1^)		Diameter of inhibition zone (mm)			
		*C. albicans*	*A. brasiliensis*	*A. alternata*	*F. oxysporum*
**Ag NPs**	0.00 (C)	0.00^e^	0.00^c^	0.00^c^	0.00^c^
	62.5	0.00^e^	0.00^c^	0.00^c^	0.00^c^
	125	14.33 ± 0.58^d^	0.00^c^	0.00^c^	0.00^c^
	250	22.00 ± 1.00^c^	0.00^c^	0.00^c^	0.00^c^
	500	27.33 ± 1.54^b^	12.33 ± 1.53^b^	14.33 ± 1.53^b^	13.00 ± 1.00^b^
	1000	32.67 ± 0.58^a^	23.33 ± 0.58^a^	20.67 ± 0.58^a^	21.33 ± 0.58^a^
**Se NPs**	0.00 (C)	0.00^d^	0.00^d^	0.00^d^	0.00^d^
	62.5	0.00^d^	0.00^d^	0.00^d^	0.00^d^
	125	14.33 ± 0.58^c^	13.33 ± 0.58^c^	0.00^d^	0.00^d^
	250	22.00 ± 1.00^b^	21.67 ± 0.58^b^	14.00 ± 1.00^c^	14.67 ± 0.58^c^
	500	28.33 ± 1.15^a^	31.33 ± 0.58^a^	22.00 ± 1.00^b^	22.67 ± 2.08^b^
	1000	32.67 ± 0.58^a^	35.00 ± 1.00^a^	25.67 ± 2.08^a^	33.33 ± 2.08^a^
**Ag-Se NPs**	0.00 (C)	0.00^f^	0.00^e^	0.00^d^	0.00^d^
	62.5	11.33 ± 0.58^e^	0.00^e^	0.00d	0.00^d^
	100	17.00 ± 1.00^d^	12.33 ± 1.53^d^	0.00^d^	0.00^d^
	250	26.33 ± 1.52^c^	17.67 ± 0.58^c^	11.33 ± 1.53^c^	12.33 ± 1.53^c^
	500	34.00 ± 2.65^b^	26.00 ± 1.00^b^	22.33 ± 1.53^b^	21.67 ± 0.58^b^
	1000	42.67 ± 1.15^a^	32.67 ± 0.58^a^	30.67 ± 0.58^a^	30.33 ± 2.08^a^
**Nystatin**		11.33 ± 0.58	0.00	0.00	0.00

*Nystatin was used at a concentration of 100 µg mL*^*− 1*^. *Calculated mean is for triplicate measurements from three independent experiments ± SD*, ^*a−e*^*means with different superscripts in the same column for each nanoparticle are considered statistically different (LSD test, P ≤ 0.05)*

Table 3 displays the antibacterial characteristics of four distinct human pathogenic bacterial strains against which the synthesized Ag NPs, Se NPs, and bimetallic Ag-Se NPs were tested. The gathered data amply illustrated the broad spectrum of antibacterial properties of all the synthesized NPs when contrasted with Amoxicillin (the gold standard antibiotic). Additional information from the collected data revealed that the reported MIC-values varied according to the kind of NPs used in the synthesis and the subject of the study bacterial species. While *P. aeruginosa* and *K. pneumoniae* were the most antibiotic-resistant organisms, the three different types of NPs were most efficient against *E. coli*. The information acquired (Table 3) additional shown that the reported MIC-values for bimetallic Ag-Se NPs against *E. coli* were 62.5 µg mL^− 1^. Ag NPs and Se NPs both had MIC values of 125 µg mL^− 1^.

**Table 3:** Antibacterial activity of Ag NPs, Se NPs, and bimetallic Ag-Se NPs synthesized by bacterial filtrate and gamma-rays against different Gram-positive and Gram-negative human pathogenic bacterial strains

NPs concentration (µg mL^− 1^)		Diameter of inhibition zone (mm)			
		*E. coli*	*S. aureus*	*P. aeruginosa*	*K. pneumoniae*
**Ag NPs**	0.00 (C)	0.00^e^	0.00^d^	0.00^c^	0.00^c^
	62.5	0.00^d^	0.00^d^	0.00^c^	0.00^c^
	125	12.67 ± 1.53^d^	0.00^d^	0.00^c^	0.00^c^
	250	18.33 ± 1.15^c^	16.33 ± 1.15^c^	0.00^c^	0.00^c^
	500	25.33 ± 1.15^b^	27.33 ± 1.15^b^	21.33 ± 0.58^b^	11.33 ± 0.58^b^
	1000	30.67 ± 0.58^a^	34.33 ± 0.58^a^	30.00 ± 1.00^a^	16.67 ± 1.15^a^
**Se NPs**	0.00 (C)	0.00^e^	0.00^d^	0.00^d^	0.00^d^
	62.5	0.00^e^	0.00^d^	0.00^d^	0.00^d^
	125	12.67 ± 1.15^c^	15.33 ± 1.15^c^	0.00^d^	0.00^d^
	250	20.67 ± 1.15^c^	21.67 ± 1.15^b^	08.67 ± 0.58^c^	11.67 ± 1.15^c^
	500	33.33 ± 2.08^b^	24.67 ± 0.58^b^	12.67 ± 0.58^b^	15.33 ± 1.15^b^
	1000	38.67 ± 0.58^a^	35.33 ± 1.15^a^	19.00 ± 1.00^a^	23.33 ± 1.52^a^
**Ag-Se NPs**	0.00 (C)	0.00^e^	0.00^f^	0.00^e^	0.00^e^
	62.5	8.67 ± 0.58^d^	7.33 ± 0.58^e^	0.00^e^	0.00^e^
	100	12.67 ± 0.58^c^	10.67 ± 0.58^d^	8.67 ± 0.58^d^	10.00 ± 1.00^d^
	250	23.33 ± 1.53^b^	14.67 ± 1.52^c^	13.67 ± 1.00^c^	13.67 ± 0.58^c^
	500	32.67 ± 1.53^a^	23.33 ± 2.51^b^	18.33 ± 2.52^b^	21.33 ± 2.08^b^
	1000	34.67 ± 2.52^a^	29.33 ± 1.52^a^	28.33 ± 2.31^a^	29.67 ± 1.53^a^
**Amoxicillin/Clavulanic acid**		9.67 ± 1.15	0.00	0.00	0.00

*Amoxicillin/Clavulanic acid was used at a concentration of 100 µg mL*^*− 1*^. *Calculated mean is for triplicate measurements from three independent experiments ± SD*, ^*a−e*^*means with different superscripts in the same column for each nanoparticle are considered statistically different (LSD test, P ≤ 0.05)*

Ag NPs have been studied in several papers as potent antibacterial agents against different infections. Ag NPs were created by suspending graphene oxide sheets, as described by Das et al. [76] after which their antibacterial activity was investigated. Their results showed that increasing the quantity of Ag NPs enhanced the development inhibition of *P. aeruginosa* and *E. coli*, indicating that Ag NP concentration is an important factor that influences their antibacterial action.

According to Saeb et al. [77] the production of biogenic Ag NPs also made use of a range of soil isolates. Further research was done on how well these antimicrobial drugs worked against isolates of MDR microbes and other highly transmissible bacteria. The pathogens *E. coli*, *S. aureus*, *S. epidermidis*, and *K. pneumoniae* were all effectively combatted by the generated Ag NPs.

Last but not least, Shepherd et al. [78] evaluated the effectiveness of the biogenic Ag NPs (75.0 ppm) towards *E. coli*, which had a ZOI of around 15.0 mm, whereas *S. aureus* was found to have a ZOI of 14.0 mm. Additionally, 11.0 and 12.0 mm ZOI towards *E. coli* and *S. aureus* were found in the active Ag^+^ ions.

After taking into consideration the small amount of Ag that helps minimize the toxic degree of the synthesized bimetallic NPs and particular combinations among Ag and Se atoms, the highest antimicrobial activity at small quantities is due to the synergistic potential between Ag and Se in the synthesized bimetallic Ag-Se NPs. These enhanced characteristics enable for the feasible application in different fields of medicine with the accurate treatment [79–81].

Rising resistance to antibiotics is a severe global health concern, making it urgently necessary to develop new antimicrobial formulations to fight drug-resistant microorganisms. Drugs that utilize NPs as antimicrobial substances have lately gained a lot of attention in the research of microbial drug resistance [82]. In order to determine the efficiency of the three types of NPs that were synthesized in this work as possible antimicrobials touching various MDR bacterial strains as well as several human and plant harmful fungi, they were investigated.

The gathered information supported the broad-spectrum antibacterial activity of all produced Ag NPs, Se NPs, and bimetallic Ag-Se NPs, which inhibited all kinds of bacteria and fungi. According to our findings, Ag NPs and Se NPs have been well-reported for their antibacterial efficacy against a number of bacterial and fungal species [83–85]. Some NPs may often damage microbial organisms by interacting with the cell wall or membrane, which can cause the leaking of genetic information, proteins, and minerals [82, 83]. Along with to their significant effectiveness in preventing cellular development by triggering cell death for increasing the formation of reactive oxygen species [86].

### Anti-biofilm potential of the synthesized NPs

Exopolysaccharides frequently form biofilms is formed in many pathogenic bacteria [46]. Using the tube approach, nutrient broth inoculations with and without the inclusion of the synthesized NPs were used to assess the development of bacterial biofilms.

Without Ag NPs, Se NPs, or bimetallic Ag-Se NPs, microbial pathogens will grow in test tubes and might produce an extensive pale yellow matt at the air-liquid interface. It was then attached to the tube walls, dyed with crystal violet (CV), and appeared as deep blue rings. A dark blue solution was created when CV was dissolved in ethanol and was utilized for subsequent semi-quantitative analyses. In contrast, the tube containing the tested microbes that had been treated with 5.0 µg/mL Ag NPs, Se NPs, and bimetallic Ag-Se NPs displayed poor growth and biofilm production when compared to the control (tube containing only the tested microbes), as shown by the lighter blue solution after CV was dissolved in ethanol.

The UV-Vis. spectrophotometer was set at 570.0 nm to measure the inhibition percentage (%) of the tested pathogens. After ethanol was used to separate the stained biofilm, the O.D. was calculated. As indicated in Table 4, the percentage of inhibition against *C. albicans* that was inhibited by 5 µg/mLAg-Se NPs was 90.88%, followed by 90.70% for *E. coli* and 90.62% for *S. aureus*. The same situation was observed for the synthesized Ag NPs with high antibiofilm potential as 87.57%, 86.71%, and 86.62% against *S. aureus, E. coli*, and *P. aeruginosa*, respectively. Finally the synthesized Se NPs were inhibit biofilm formation in *C. albicans* (86.39%), *E. coli* (82.09%), and *S. aureus* (76.04%). It must be noted that, the combination between Se and Ag NPs as bimetallic Ag-Se NPs increasing the antibiofilm potential as seen in Table 4.

**Table 4:** Semi-quantitative inhibition % of the biofilm formation for non-treated and treated bacterial and yeast pathogens with Ag NPs, Se NPs, and bimetallic Ag-Se NPs

*Test organism*	O.D. of crystal violet stain at 570.0 nm				Inhibition %		
	**Control**	**Treated with Ag NPs**	**Treated with Se NPs**	**Treated with Ag-Se NPs**	**Ag NPs**	**Se NPs**	**Ag-Se NPs**
***E. coli***	0.888^c^ ± 0.008	0.118^c^ ± 0.002	0.159^d^ ± 0.002	0.109^d^ ± 0.009	86.71	82.09	87.70
*** S. aureus***	0.789^d^ ± 0.006	0.098^d^ ± 0.003	0.189^c^ ± 0.001	0.074^b^ ± 0.009	87.57	76.04	90.62
***P. aeruginosa***	0.912^a^ ± 0.007	0.122^b^ ± 0.005	0.280^b^ ± 0.005	0.089^c^ ± 0.009	86.62	69.29	90.24
*** K. pneumoniae***	0.891^b^ ± 0.002	0.389^a^ ± 0.006	0.445^a^ ± 0.006	0.198^e^ ± 0.009	56.34	50.72	77.77
*** C. albicans***	0.669^e^ ± 0.004	0.092^d^ ± 0.003	0.091^e^ ± 0.003	0.061^a^ ± 0.009	86.42	86.39	90.88

Values are means  ±  SD (n = 3). Data within the groups are analyzed using one-way analysis of variance (ANOVA) followed by ^a, b, c, d, e^ Duncan’s multiple range test (DMRT)

The study of biofilm inhibition is continually developing, and the factors involved in biofilm development have been discovered and are being investigated as possible therapeutic targets [46]. Still, further research is needed about the agents that prevent infections from building biofilms. Wood et al., [87], discovered that several non-toxic anti-biofilm drugs, including indole compounds, 5-fluorouracil, and ursolic acid, interacted with *E. coli*.

It is still unclear what exactly causes resistance to bacterial biofilms. The creation of glycocalyx, a pericellular matrix made up of glycolipid and glycoprotein, which improves bacterial biofilms and reduces the impact of other antibacterial drugs while decreasing patient resistance, is an intriguing discovery [88].

Antibacterial drugs are used to prevent the formation of bacterial biofilms by obstructing polysaccharide layers, which therefore makes it easier to restrict the growth of bacterial cells. The advancement of nanotechnology allows for the quick manufacture of particles and nanocomposite with effective antibiofilms [89, 90].

Exopolysaccharide formation, which is crucial for the formation of biofilms, was visible in the non-treated pathogens with Ag NPs, Se NPs, and bimetallic Ag-Se NPs. However, after treatment with Ag NPs, Se NPs, and bimetallic Ag-Se NPs, these pathogens were significantly inhibited. Thus, pathogens are prevented from forming biofilms by impeding the production of exopolysaccharides. Similar outcomes against *S. epidermidis* and *P. aeruginosa* biofilms were also seen by Kalishwaralal et al. [91] who found that 100 nM of the produced Ag NPs provided 96–99% of biofilm suppression.

The synthesized Ag NPs, Se NPs, and bimetallic Ag-Se NPs (in this study) exhibit antibiofilm inhibition % in a suitable and encouraging result at low concentrations, as compared to those in the scientific literature that have antibiofilm potential.

According to Kasi Gopinath et al.’s antibiofilm findings [92], all of the tested microorganisms had inadequate adhesion and disconnected biofilm external architecture after being incubated overnight for biofilm growth. The antibiofilm properties of bimetallic Ag-Se NPs showed more notable restraint of biofilm mass width as a result of the combined benefits of Ag and Se in the synthesized bimetallic Ag-Se NPs. Not least, our results were related to recently published research [93–96].

According to earlier studies, Ag NPs can considerably lower the amount of bacterial cells, which in turn lowers the capacity to form biofilms. Some synthesized Ag-Au NPs were able to penetrate bacterial cell walls and their surfaces. Additionally, in our earlier research, several synthesized metal NPs’ possible modes of action against particular infections were described [58, 97].

### DPPH free radical scavenging activity of the synthesized Ag NPs, Se NPs and bimetallic Ag-Se NPs

All three NPs shown promising antioxidant activity at various concentrations, according to the results of contrasting the antioxidant behavior of synthesized Ag NPs, Se NPs, and bimetallic Ag-Se NPs with ascorbic acid (Table 5). Ascorbic acid and the three different kinds of the synthesized NPs had the lowest inhibitory levels at 25 µg mL^− 1^, according to the results. The observed outcomes (Table 5) further demonstrated that all three types of the synthesized NPs reduced DPPH free radicals in a dependent on dose way, with any rise in the administered concentration of NPs creating a significant (P ≤ 0.05) increase in the observed scavenging activity.

**Table 5:** DPPH free radical scavenging activity of Ag NPs, Se NPs, and bimetallic Ag-Se NPs

NPs concentration (µg mL^− 1^)	Free radical scavenging activity (%)			
	Ascorbic acid	Ag NPs	Se NPs	Ag-Se NPs
**0.00 (C)**	0.00^ g^	0.00^ h^	0.00^ h^	0.00^ g^
**25**	20.56 ± 2.56^f^	15.11 ± 1.36^ g^	17.21 ± 1.45^ g^	19.32 ± 2.56^f^
**50**	47.65 ± 3.45^e^	27.87 ± 1.41^f^	36.76 ± 3.54^f^	38.53 ± 5.15^e^
**100**	55.43 ± 4.87^d^	38.67 ± 4.52^e^	51.81 ± 2.61^e^	50.32 ± 3.64^d^
**200**	61.08 ± 6.33^c^	48.56 ± 4.87^d^	59.59 ± 5.44^d^	58.56 ± 1.56^c^
**400**	75.22 ± 8.51^b^	62.06 ± 3.56^c^	68.54 ± 5.42^c^	68.65 ± 4.48^b^
**800**	98.71 ± 6.32^a^	75.17 ± 2.66^b^	74.32 ± 7.06^b^	89.34 ± 3.42^a^
**1000**	100.00 ± 0.00^a^	83.65 ± 7.51^a^	82.41 ± 6.11^a^	91.14 ± 7.85^a^
**IC**_**50**_ **(µg mL** ^**− 1**^ **)**	71.61	242.54	124.53	98.52

*DPPH scavenging assay was used for measuring the antioxidant activities of the synthesized nanoparticles at 517 nm using DPPH solution under the conditions described in Materials and Methods. Calculated mean is for triplicate measurements from three independent experiments ± SD*, ^*a−h*^*means with different superscripts in the same column are considered statistically different (LSD test, P ≤ 0.05)*

The results that were obtained further demonstrated that the recorded IC50-values for bimetallic Ag-Se NPs, Se NPs, and Ag NPs were 98.52 µg mL^− 1^, 124.53 µg mL^− 1^, and 242.54 µg mL^− 1^, respectively. Ascorbic acid’s IC50 measurement was found to be 71.61 µg mL^− 1^. As a result, the synthesized NPs may be sorted as follows in decreasing order of antioxidant potential: Ag-Se NPs > Se NPs > Ag NPs.

When compared to ascorbic acid, the synthesized Ag NPs, Se NPs, and bimetallic Ag-Se NPs showed promising antioxidant potential in the current study, according to the findings of the evaluation of their antioxidant activity. Metal nanoparticles’ capacity to neutralize free radicals has been extensively documented in the literature [48, 85, 98]. The antioxidant effect of the metal NPs was primarily caused by the movement of electrons, which neutralized and stopped the DPPH from producing free radicals [99]. Additionally, a high surface-to-volume ratio may boost the antioxidant activity of metal nanoparticles [100]. According to literature findings, Se NPs produced by *Monascus purpureus* culture extract had equal antioxidant activity to ascorbic acid in a dose-dependent manner at a concentration of 85.92 µg mL^− 1^ [85]. When compared to ascorbic acid, Se NPs produced by *Withania somnifera* leaf extract shown modest antioxidant activity in a dose-dependent manner at concentrations between 20 µg mL^− 1^ and 100 µg mL^− 1^ [84]. As a result, the antioxidant activity seen in our work is encouraging in terms of concentration and offers a direction for further investigation of bimetallic Ag-Se NPs as a potential new source of antioxidants.

### Photocatalytic activity of Ag NPs, Se NPs, and bimetallic Ag-Se NPs

**Fig. 7:**
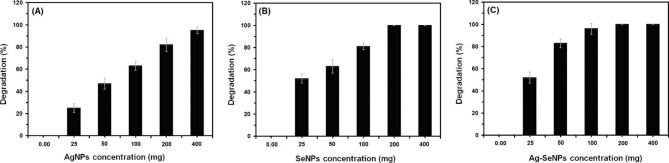
Photocatalytic degradation of MB dye by **(A)** Ag NPs, **(B)** Se NPs, and **(C)** bimetallic Ag-Se NPs

The photocatalytic activity of the synthesized Ag NPs, Se NPs, and bimetallic Ag-Se NPs was evaluated by the degradation of MB dye after they had been left in the dark for 1 h to attain equilibrium. The fact that the dye degradation occurred in the presence of the synthesized Ag NPs, Se NPs, and bimetallic Ag-Se NPs was confirmed by a reduction in the measured absorbance (at 664 nm) following twenty minutes of being exposed to sunlight. Deterioration was demonstrated by the MB’s gradual change in color from a dark blue to colorless. The impact of various Ag NPs, Se NPs, and bimetallic Ag-Se NP concentrations on the degradation of MB dye was shown in Fig. 6. The acquired data, as can be seen in Fig. 7, demonstrated that the three distinct types of NPs successfully obliterated the MB dye in a concentration-dependent way. The proportion of degradation steadily rises with a rise in either the quantity of Ag NPs, Se NPs, or bimetallic Ag-Se NPs. Complete degradation (100%) was demonstrated for Se NPs and bimetallic Ag-Se NPs both at a concentration of 200 mg; yet, for Ag NPs, it occurred at a concentration of 400 mg.

In this investigation, dye degradation of MB during 20 min of sunshine was used to assess the photocatalytic behavior of the synthesized Ag NPs, Se NPs, and bimetallic Ag-Se NPs. The MB dye was successfully broken down by the synthesized NPs in a concentration-dependent manner, with 200 mg being the magic number for both Se NPs and bimetallic Ag-Se NPs. Recently, the study of catalytic activation of industrial wastes has given metallic nanoparticles a lot of attention [18, 101, 102]. Data on the photocatalytic activity of NPs produced by microorganisms are few in the literature. However, no papers have examined the photocatalytic behavior of bimetallic Ag-Se NPs produced by microorganisms in the literature.

The photocatalytic activity of ZnO NPs produced by *Alternaria tenuissima* was investigated by Abdelhakim et al. [48]. Under UV irradiation, ZnO NPs enabled complete breakdown (100%) of the MB dye. Furthermore, according to Kalpana et al. [103], Bismarck brown dye showed greatest degradation (89%) when used with 100 µL ZnO NPs when exposed to UV radiation for 72 h.

Up to present deep discussion, the synthesized bimetallic Ag-Se NPs created in this work shown excellent efficiency in the MB degrading process. Consequently, they can be used in the textile and water treatment sectors.

## Conclusion

This paper proposed a novel approach to create bimetallic Ag-Se NPs, Se NPs, and Ag NPs from bacterial filtrate in an environment of gamma rays. According to HR-TEM imaging, the synthesized Ag NPs, Se NPs, and bimetallic Ag-Se NPs had a variety of round, sometimes irregular, and oval morphologies. Analyses of the form, crystallinity, and distribution underwent thorough confirmation. Se NPs had a median diameter of 40.7 nm with sizes ranging from 22.4 to 98.1 nm. The synthesized bimetallic Ag-Se NPs had an average diameter of 46.7 nm with a size range of 38.2 to 59.3 nm. Last but not least, Ag NPs varied in diameter by 34.3 nm and ranged in size from 26.2 to 50.5 nm. An argument for the impending and ongoing reduction in ions brought on by the effects of the gamma rays at 20.0 kGy and 15 kGy. Antimicrobial activity against several pathogenic bacteria, fungi, and yeast was studied using ZOI and MIC methods. Ag-Se NPs (at low concentrations) inhibited the growth and biofilm formation of *C. albicans*, *E. coli*, and *S. aureus* in percentages of 90.88%, 90.70%, and 90.62%, respectively. This study is scientifically sound due to the generated NPs’ high stability for an extended period due to the bacterial filtrate’s capping ability and their potential antibacterial properties at low amounts, which raised the potential for the likely application in long-term goals. The prepared bimetallic Ag-Se NPs have the potential to be useful in a variety of pharmaceutical, environmental, and healthcare uses as well as to their contribution to the breakdown of MB dye via their potential as an effective photocatalyst, particularly as antimicrobial substances towards some infectious bacteria and announcing antioxidants.

## Data Availability

The datasets used and/or analyzed during the current study are available from the corresponding author on reasonable request.
